# Clinical significance of Polycomb gene expression in brain tumors

**DOI:** 10.1186/1476-4598-9-265

**Published:** 2010-09-30

**Authors:** Francesco Crea, Elaine M Hurt, William L Farrar

**Affiliations:** 1 Cancer Stem Cell Section, Laboratory of Cancer Prevention, National Cancer Institute at Frederick, Center for Cancer Research, National Cancer Institute, Frederick, MD, USA; 2 Scuola Superiore Sant'Anna, Pisa, Italy

## Abstract

Polycomb group (PcG) proteins are crucial for neural cancer stem cell (NCSC) self-renewal. However, the relative expression levels of PcG genes in different subtypes of brain tumors, their prognostic role and their effects on cellular pathways have not been investigated. For this purpose, we queried the Oncomine database and found that 4 PcG genes (EZH2, RBBP7, SUZ12, YY1) are specifically expressed in brain tumors. EZH2 expression increases with tumor grade in adult and pediatric brain tumors, and is a poor prognostic factor. In glioblastoma, EZH2 inhibits differentiation, and activates cancer-, cell cycle- and cellular movement-related genes. In keeping with previously published data, our results suggest that EZH2 is both a prognostic factor and a promising therapy target in brain tumors.

## Findings

Central Nervous System (CNS) tumors occur at each stage of life, and are therefore classified as embryonal, pediatric and adult cancers [1-3]. Treatment and prognosis are dependent on tumor histology. For pediatric and adult CNS cancers, tumor grade is the main prognostic factor [1,3].

Due to the lack of effective therapies for aggressive CNS tumors, the identification of new targets and prognostic indicators is warranted. Recent evidence shows that, in addition to genetic changes, CNS tumors are driven by epigenetic alterations, namely DNA methylation and histone post-translational modifications [4]. Polycomb group (PcG) genes are epigenetic effectors involved in CNS development and cancer progression [5]. PcG proteins are organized in polycomb repressive complexes (PRCs). During development, PRCs catalyze histone post-translational modifications and gene silencing. The best characterized complexes are PRC1 and PRC2. The latter mediates histone H3K27 methylation. PRC1 binds to this chromatin modification and catalyzes histone H2A ubiquitylation. PRCs silence lineage-specific genes in embryonic and adult stem cells (SCs) [5]. During brain development, PRCs are expressed in a time- and region-specific manner, thereby orchestrating SC proliferation and differentiation [6]. Each PRC isoform targets a different set of *loci*. This combinatorial complexity affects the subtle balance between SC self-renewal and differentiation [5].

Recent evidence indicates that most CNS tumors are driven by a small population of CD133^+ ^neural cancer stem cells (NCSC) [7]. These cells are resistant to conventional chemotherapy [8,9] and are the only cells able to initiate a tumor when injected into immunocompromised mice [7]. In adition, CD133 is up-regulated in high grade CNS tumors, and is a poor prognostic indicator [10]. Targeting NCSCs could eradicate CNS tumors [11,12]. However, most data in favor of the NCSC hypothesis are derived from mouse and *in vitro *studies. The role of NCSC in the clinical setting is still elusive.

For their function in SC biology and cancer [5], PcG genes are obvious candidates for NCSC-specific targeted therapy. PcG targets are specifically silenced in brain tumors [13]. and the PRC2 member EZH2 may be overxpressed in gliomas [14]. In addition, BMI1 copy number alteration is frequent in human gliomas [15]. BMI1 is a PRC1 component, essential for NCSC self-renewal and tumorigenicity [16]. PcG genes are also involved in CD133^+ ^glioma SC radioresistance [17].

In the present paper, we queried the Oncomine database to systematically assess relative gene expression levels of PcG genes in CNS tumors. Gene expression data from embryonic, pediatric and adult brain tumors were collected from Oncomine database http://www.oncomine.com. Data were from 34 independent studies. We investigated gene expression profile of 21 PcG genes: Pc, PH, RING and PSC homologs (PRC1); EED, EZH2, SUZ12 (PRC2); YY (1 and 2), SCML1, SIRT, L3MBTL2, RBBP. These genes have been selected based on previously published lists [18,19]. We compared gene expression in normal brain vs. cancer tissues, and in different histological subtypes.

To identify PcG targets inactivated in brain tumors, we interrogated the following Oncomine categories: "PcG target genes in human embryonic SCs" and "Top 10% downregulated genes in glioblastoma (Sun Brain)". We found 106 overlapping genes. Since these genes were identified by the intersection of data from genes that are silenced by PRC2 in human embryonic SCs and from glioblastoma studies, these 106 overlapping genes will be hereinafter called "PRC2 targets in brain tumors".

To identify genes expressed in association with EZH2, coexpression data from 3 large studies (Beroukhim, Phillips, Sun, 467 patients) were collected. We found 213 genes positively correlated to EZH2 (R > 0.70). These genes will be called "EZH2-associated genes".

All statistical values relative to this meta-analysis were calculated as described by Chinnaiyan and colleagues [20].

**Figure 1:**
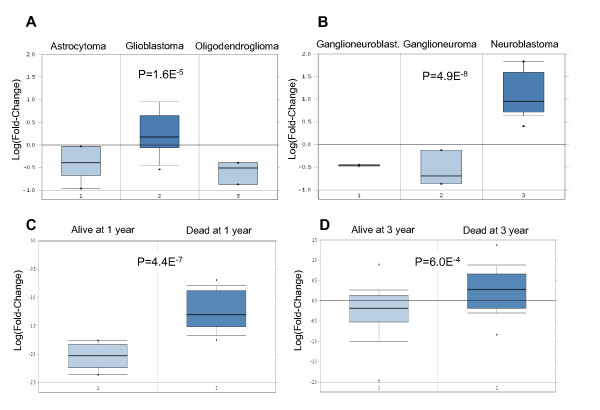
**EZH2 expression in neurological tumors**. A, B: EZH2 is overexpressed in higher grade tumors. C, D: EZH2 overexpression is associated to poor prognosis in human glioblastoma (Oncomine database).

Table 1 summarizes differentially expressed PcG genes in normal brain, brain tumors and specific tumor histologies. Surprisingly, some PRC1 components (BMI1, CBX2, CBX7) were downreglated in glioblastoma compared to normal brain. Other PRC1 members (CBX7, PCGF6) were selectively expressed in lower grade gliomas. In contrast, the PRC2 components EZH2 and SUZ12 were always overexpressed in tumors compared to normal neural tissue. In addition, these 3 independent studies all showed a highly significant correlation between EZH2 overexpression and gliomas. Moreover, EZH2 expression significantly increased with tumor grade in both adult and pediatric brain tumors (Figure 1A, B).

**Table 1:** Polycomb genes expression in normal brain and specific tumor histologies.

PRC	GENE	Category 1 (up-regulated)	Category 2 (down- regulated)	P value
1	BMI1	Normal Brain	Glioblastoma	5.85 E-27
	CBX2	Neural Stem Cell	Glioblastoma	4.4 E-13
	CBX7	Normal Brain	Glioblastoma	2.2 E-27
		Ganglioneur-oma/-oblastoma	Neuroblastoma	2.4 E-7
	PCGF1	Glioblastoma	Astrocytoma	1.2 E-6
	PCGF6	Astrocytoma/Oligodendroglioma	Glioblastoma	4.0 E-6
	PHC2	Astrocytoma/Glioblastoma	Oligodendroglioma	1.4 E-7
	YY1	Anaplastic Oligoastrocytoma	Normal Brain	1.23 E-5
		Medulloblastoma with Advanced Neural Differentiation	Other hystologies	8.86 E-27
		Oligodendroglioma	Mixed Glioma	3.83 E-6

2, 3, 4	EZH2	Anaplastic Oligodendroglioma	Normal Brain	1,12E-12
		Anaplastic Astrocytoma	Normal Brain	3,50E-13
		Glioblastoma	Normal Brain	2,56E-23
		Neuroblastoma	Ganglioneur-oma/-oblastoma	4,94E-08
		Glioblastoma	Astrocytoma/Ogligodendroglioma	1.64 E-5
	RBBP7	Anaplastic Astrocytoma	Normal Brain	6,0E-06
		Anaplastic Oligodenroglioma	Normal Brain	5,60E-08
	SUZ 12	Anaplastic Oligodendroglioma	Normal Brain	1.19 E-7
		Atypical Theratoid/Rhabdoid Tumor	Medulloblastoma	5.50 E-8

4	SIRT1	Neural Stem Cell	Glioblastoma	9.4 E-13
		Oligodendroglioma	Glioblastoma Astrocytoma	1.9 E-11

Data derived from Oncomine database. Grading analyses were performed on 35 adult and 19 pediatric brain tumors. Survival analysis was performed on a total of 97 glioblastoma patients.

These results show that PRC2 genes may play a crucial role in adult and pediatric brain tumors. BMI1 mRNA expression seems not to predict tumor progression. This seems to be in contrast with previous evidence [16], showing that BMI1 protein sustains glioblastoma CSC self-renewal. However, BMI1 protein and mRNA levels may not be directly correlated, and many post-transcriptional modulations may explain this discrepancy.

**Figure 2:**
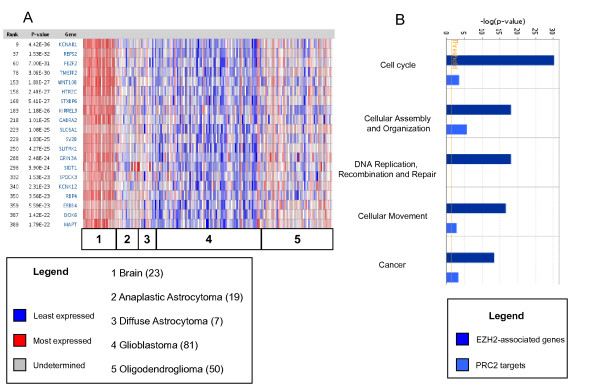
**Polycomb-related gene expression in glioblastomas**. A: PRC2 target genes are selectively silenced in glioblastomas (Oncomine database). B: Functional analysis comparing EZH2-associated genes (upper column, dark blue) with Polycomb targets (lower column, light blue) in glioblastomas (IPA software).

Since EZH2 is essential for NCSC self-renewal [12], and due to our results showing that EZH2 is overexpressed in brain tumors, we investigated the prognostic role of EZH2 in gliobalstoma. Two independent studies showed a highly significant association between EZH2 overexpression and poorer 1 year- and 3 year-survival in glioblastoma patients (Figure 1C, D). We identified a significant and progressive (p = 4.4E^-7^, odds ratio = 2.0) pattern of PRC2 target gene silencing in gliomas (Figure 2A). In addition, a list of 52 PRC2 targets was downregulated in poor prognosis-glioblastoma patients, and predicted shorter 3-year survival (p = 4.2E^-6^, odds ratio = 2.2, additional file 1).

Our analysis suggests that glioma progression is associated with EZH2 overexpression and PRC2 target gene silencing. Thus, we investigated which pathways are co-activated with EZH2, and which pathways are silenced by PRC2. For these purposes, we compared two sets of genes: PRC2 targets in glioma and EZH2-associated genes. The latter category includes 213 genes positively correlated with EZH2, identified in three large microarray studies in brain tumors. Ingenuity pathway analysis (IPA, Ingenuity^® ^Systems, http://www.ingenuity.com of EZH2-associated genes showed the following top categories: "Cancer" (4.6E^-14^<p < 1.5E^-2^), "Cell cycle" (6.02E^-31^<p < 1.5E^-2^) and "Embryonic development" (2.3E^-5^<p < 1.5E^-2^). Interestingly, when we compared IPA for both EZH2-associated genes and PRC2 targets in brain tumors, we found that the first gene set was significantly enriched for Cell-cycle- Cancer-, and Cellular movement-associated genes, compared to PRC2 targets (Figure 2B). Gene categories significantly enriched in PRC2 target genes included: "Behavior", "Neurological disorders" and "Psychological disorders". Our analysis suggests that PRC2 specifically silences genes involved in mature brain functions, and that EZH2 is overexpressed in undifferentiated cells.

**Figure 3:**
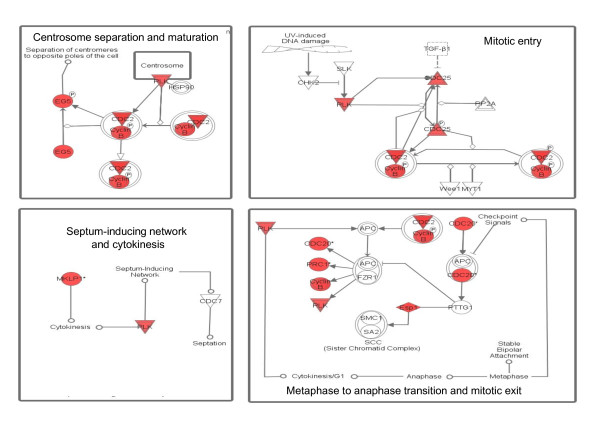
**Mitotic roles of polo-like kinases**. EZH2 associated genes (in red) are highly represented in all mitotic pathways involving polo-like kinases. Data obtained through IPA software.

In addition, EZH2 may indirectly activate cell cycle progression, tumorigenesis and invasion, as shown in other cancer types [5]. In this regard, the top canonical pathway activated in EZH2-associated genes was "Mitotic roles of Polo-like kinases" (p = 1.83E^-11^). As shown in Figure 3, several EZH2-associated genes are involved in this pathway, which regulates mitotic entry and DNA damage-activated checkpoints. Interestingly, Polo kinases orchestrate the self-renewal versus differentiation decision in neural progenitors, by regulating the orientation of mitotic spindles [21]. Additional pathways significantly associated with EZH2 expression in glioma were "ATM signaling" (p = 2.2E^-6^), "G2/M DNA damage checkpoint" (p = 1.0E^-5^) and "Sonic hedgehog signaling" (p = 1.5E^-3^). ATM signaling is involved in DNA repair, while Hedgehog pathway is essential for neural SCs [5]. Our results are in agreement with in vitro findings, showing that EZH2 is required for cell cycle control and SC self-renewal [5].

To corroborate our findings, we uploaded PRC2 target genes and EZH2-associated genes on the Broad Institute's Molecular Signature Database http://www.broadinstitute.org/gsea/msigdb/annotate.jsp, and compared significant overlaps with gene ontology signatures. EZH2-associated genes were significantly correlated with "Spindle" (p = 7.6E^-15^) and "Mitotic cell-cycle" (p = 8.6E^-15^). These categories resembled IPA "Cell Cycle" sub-categories correlated to EZH2: "Spindle Checkpoint" (p = 5.3E^-11^) and "M-phase of eukaryotic cells" (p = 1.2E^-15^)^. ^PRC2 target genes were associated to "Trasmission of nerve impulse" (p = 9.1E^-5^) and "Axonogenesis" (p = 1.6E^-4^). These results corroborate the hypothesis that PRC2 inhibits neural differentiation-associated genes in human brain tumors. In addition, they confirmed the likelihood of a relationship between EZH2 and mitotic control. Interestingly, EZH2-associated genes were also significantly linked to "Up-regulated in doxorubicine resistant cells" (p = 2.2E^-36^). This again highlights the probability of an association between EZH2 expression and NCSCs, since these are the cells thought to be responsible for chemotherapeutic resistance.

In conclusion, we found a correlation between PRC2-mediated gene silencing and brain tumor progression. In particular, EZH2 predicts poor prognosis higher grade. Network analysis revealed that brain tumor cells overexpressing EZH2 display activation of SC- and cell cycle-specific pathways, and consistent with this data, differentiation genes are silenced by PRC2. Our results obtained on patient gene expression profiles corroborate in vitro findings, showing that PRC2 is required for NCSC self-renewal and for glioblastoma tumorigenesis [12]. Interestingly, 3-dezaneplanocin A (DZNeP) was shown to disrupt PRC2 activity, thereby targeting NCSC tumorigenicity [12].. Our findings suggest that PRC2 is a viable target for human glioma and possibly for other neurological tumors. Indeed, we found that EZH2 expression progressively increases with pediatric tumor grade (figure 1B). Finally, we identified pathways associated with EZH2 expression in brain tumors. Along with classical cell cycle and SC pathways, we found a correlation between EZH2 and polo-like kinases. Since polo kinases orchestrate symmetric division in neural SC [21], this new link is potentially informative. Polo-like kinase 1 is overexpressed in human gliomas [22], and specific inhibitors of these kinases are being investigated as anticancer agents [23]. Thus, our results can pave the way to the rational development of treatments that specifically target NCSC. These drug regimens may include PRC2 and polo-like kinase inhibitors.

## List of abbreviations

CNS: Central Nervous System; K: Lysine; IPA: Ingenuity pathway analysis; NCSC: Neural cancer stem cell; PcG: Polycomb group; PRC: Polycomb repressive complex; SC: Stem Cell

## Competing interests

The authors declare that they have no competing interests.

## Authors' contributions

FC performed the analysis and drafted the manuscript. EH helped in manuscript drafting and supervised analysis methodology. WLF conceived the study and helped in manuscript drafting. All authors read and approved the final manuscript.

## Supplementary Material

Additional file 1 **List of 52 PRC2 targets that are down-regulated in poor prognosis PC patients**. Data were generated through Oncomine database. Click here for file
